# Tracking the Transport of Silver Nanoparticles in Soil: a Saturated Column Experiment

**DOI:** 10.1007/s11270-018-3985-9

**Published:** 2018-10-01

**Authors:** Karrar N. M. Mahdi, Ruud Peters, Martine van der Ploeg, Coen Ritsema, Violette Geissen

**Affiliations:** 1 Soil physics and land management group, Wageningen University and research, Wageningen, The Netherlands; 2 College of Engineering, University of Baghdad, Baghdad, Iraq; 3 RIKILT Wageningen University and Research, Wageningen, The Netherlands

**Keywords:** Silver nanoparticles, Transport, Soil, Leaching

## Abstract

Silver nanoparticles (AgNPs) can enter the environment when released from products containing them. As AgNPs enter soil, they are often retained in the soil profile and/or leached to the groundwater. This research assessed the transport of AgNPs in their “particle form” through the soil profile using a series of columns. Three soil types were put into soil columns: LSH (loam with high organic matter (OM)), LSL (loam with low OM), and Sand (no OM). The results showed that AgNP transport and retention in soil as well as particle size changes are affected by soil organic matter (OM) and the cation exchange capacity (CEC) of soil. OM affected the transport and retention of AgNPs. This was evident in the LSH columns where the OM concentration was the highest and the AgNP content the lowest in the soil layers and in the effluent water. The highest transported AgNP content was detected in the Sand columns where OM was the lowest. CEC had an impact on the particle size of the AgNPs that were retained in the soil layers. This was clear in columns packed with high CEC-containing soils (LSL and LSH) where AgNP particle size decreased more substantially than in the columns packed with sand. However, the decrease in AgNP sizes in the effluent water was less than the decrease in particle size of AgNPs transported through but retained in the soil. This means that the AgNPs that reached the effluent were transported directly from the first layer through the soil macropores. This work highlights the ability to track AgNPs at low concentrations (50 μg kg^−1^) and monitor the changes in particle size potential as the particles leach through soil all of which increases our knowledge about AgNP transport mechanisms in porous media.

## Introduction

Silver nanoparticles (AgNPs) are a metallic nanomaterial consisting of spherically shaped particles ranging in size from 1 to 100 nm (Helmlinger et al. 2016). AgNP production is growing fast and thus, so is the production of products and the development of applications that incorporate AgNPs (López-Serrano et al. 2014). Previous studies have researched the potential toxicity of AgNPs to humans and the environment (EC 2017). The increase in the production of AgNPs and the number of applications that use these AgNPs has raised concerns about the potential release of these materials into the environment during production, use, and inevitable disposal (Gottschalk and Nowack 2011; Yu et al. 2013).

AgNPs enter the soil environment through several different pathways including natural processes, like biological and chemical reduction of ionic silver, as well as anthropogenic activities, which seem to be the most significant sources (Yin et al. 2015b). AgNPs can reach the soil through plant strengthening agents (Lorenz et al. 2011; Prasad et al. 2014; Thuesombat et al. 2014), slag from incinerators (Holder et al. 2013), and contaminated sludge from sewage treatment plants (Lombi et al. 2013). In Germany, around 30% of the annual sewage sludge solids produced (2 million tons per year) from municipal wastewater treatment plants are used for farmland application (Schlich et al. 2013a). Similar amounts are used in Spain, Portugal, France, and the UK (Wiechmann et al. 2012). Furthermore, previous studies have shown that there is a large variation in the concentration and the particle size of the AgNPs that are used in consumer products which means that the different particle sizes and concentrations of AgNPs released into soil and water have different toxic effects. For example, the particle size of AgNPs used in a nasal spray is 28.9 nm at a concentration of 10 mg L^−1^ while an antifungal spray has AgNP with a particle size of 15.4 nm and a concentration of 30.2 mg L^−1^ (Guo et al. 2015). Once AgNPs are introduced to the soil environment, the AgNPs can be transported to deeper layers in the soil and to the groundwater (Sagee et al. 2012; Cornelis et al. 2013; Liang et al. 2013a) as well as transported with surface water runoff (Kaegi et al. 2010; Tian 2010; Mahdi et al. 2017).

AgNPs that are released to the soil environment may encounter changes in physical-chemical properties as they interact with the soil (Yin et al. 2015a). Therefore, soil characteristics can affect AgNP behavior in soil. For example, the presence of organic matter (OM) is believed to prevent AgNPs from dissolution as they coat the AgNP particles which inhibits the release of Ag^+^ (Klitzke et al. 2014) and can make the AgNPs less mobile in the soil (Coutris et al. 2012). Other studies have shown that the retention of nanomaterials generally increases, and the bioavailability decreases in soils with a finer grain size distribution (Shoults-Wilson et al. 2011a; Cornelis et al. 2012). The cation exchange capacity (CEC), ionic strength (IS), pH, and soil particle size distribution also have an impact on the fate and transport pattern of AgNPs in soil (Lowry et al. 2010; Liang et al. 2013a). Therefore, the complexity of the soil system makes it difficult to understand the transport and distribution of AgNPs in soils (Pan and Xing 2012).

In relation to toxicity, many studies have shown that smaller particle sizes of AgNPs result in higher toxicity effects (Pal et al. 2007; Kim et al. 2012; Silva et al. 2014). Furthermore, the AgNPs that are applied in a pristine form or in a transformed form (due to interactions with other soil chemicals) have both proven to be toxic to soil species (Zhang et al. 2012; Schlich et al. 2013b). Schlich et al. (2013b) also showed that AgNPs applied to soil at low concentrations could be toxic to soil microorganisms.

Detection of AgNPs in soil is complicated and requires very sensitive instruments and careful methods (Yu et al. 2015). Generally, Ag concentration in soil has been determined via the acidic digestion of the soil samples (EPA 1996). However, since AgNPs dissolve in this procedure, any information about the presence of AgNPs in the soil sample is lost and nothing can be concluded about the stability, transportation, and transformation of AgNPs in soil.

This study focused on tracking the transport and the changes in particle size of AgNPs, as they moved through 16-cm-long columns filled with different soils and in the effluent eventually leached from these columns. We used the saturated soil column method modified to mimic the real-world application of sludge-containing AgNPs and possible leaching scenarios. AgNP transport in soil increases with the increased application of rainwater (RW); therefore, RW was applied in periods of 24, 48, and 72 h in an attempt to observe the transport pattern of AgNPs in the soil and effluent water (EW) that was sampled after each RW application. The detection and characterization of AgNPs in soil was achieved using a combination of an aqueous extraction method and the single particle inductively coupled plasma mass spectrometry method (spICP-MS). This combination had the capability of detecting very low AgNP concentrations in the soil (Peters et al. 2015; Mahdi et al. 2016). The results obtained from this work contribute to filling in the knowledge gap regarding AgNP transport and leaching through soil and increases the awareness of AgNP distribution patterns in soil. These findings can be used to improve understanding and aid in the management of products and waste containing AgNPs.

## Materials and Methods

### Materials

#### Soils and Rainwater

Three types of soil were used in this study and were provided by Unifarm (Wageningen, the Netherlands). The soils used had the following characteristics: loam with high organic matter (LSH), loam with low organic matter (LSL), and sand with no OM content (Sand). The initial characteristics of the soil are given in Table 1.

**Table 1:** Soil initial properties

Characteristic		LSH	LSL	Sand
Clay (%)		10.75	9.22	0.00
Silt (%)		41.81	43.15	1.31
Sand (%)		47.45	47.64	98.69
pH (KCl)		7.5 ± 0.02	6.8 ± 0.03	7.9 ± 0.21
CEC	Ca^+2^ (meq 100^−1^)	3.8 ± 0.22	1.5 ± 0.11	0.4 ± 0.03
	K^+^ (meq 100^−1^)	0.24 ± 0.12	0.1 ± 0.12	0.02 ± 0.02
OM (%)		3.4 ± 0.46	1.8 ± 0.11	0.2 ± 0.05
IS (mM)		9.5 ± 0.60	11.9 ± 0.90	6.2 ± 0.5

*LSH* loamy soil with high organic matter content, *LSL* loamy soil with low organic matter content, *OM* organic matter, *IS* ionic strength, *CEC* cation exchange capacity.

The simulated rain was applied via a sprinkler on the top of the soil column. The rainwater setup is described in detail in the experimental design section (2.2.1). The pH and IS of the water were measured and were 7.63 and 3.8 mM respectively.

#### Chemicals

##### AgNPs

The AgNPs used consisted of an aqueous suspension of citrate-stabilized spherical AgNPs from NanoComposix (Prague, Czech Republic). The particle diameter was 60 nm and the particle mass concentration in the aqueous suspension was 1000 mg L^−1^.

##### Potassium Bromide

A potassium bromide (KBr) solution with a concentration of 0.0167 M was purchased from Fisher Scientific (Landsmeer, the Netherlands). KBr was used as an inert tracer in the experiments.

### Experimental Method

**Fig. 1:**
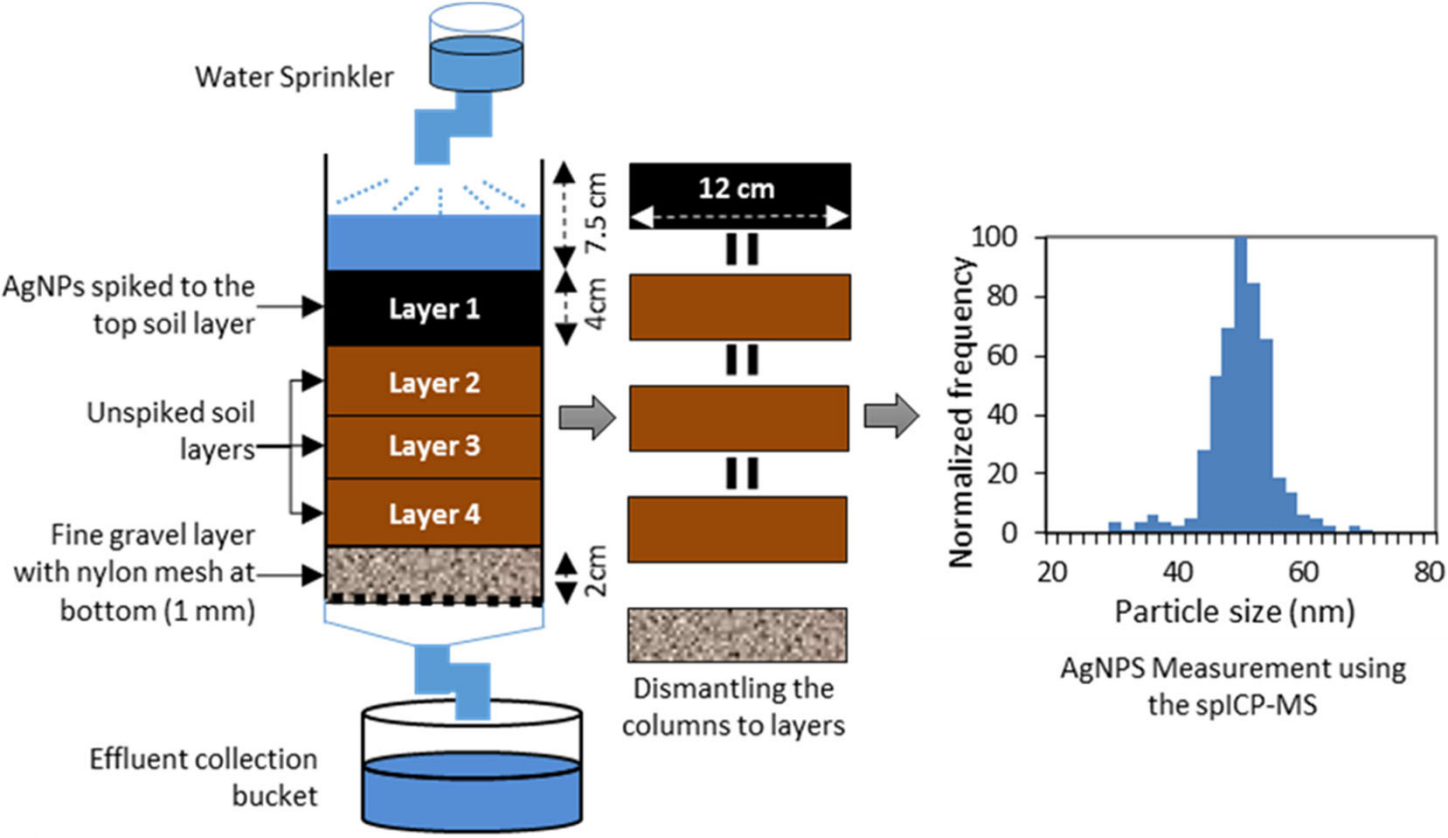
The experimental design of the column experiments

This column experiment was conducted in the soil physics laboratory of Wageningen University in the Netherlands. There were three replicates for each soil type and time period (24, 48, and 72 h), resulting in a total of 27 columns. Analyses of samples collected from the soil columns and leachate (effluent water) was performed at RIKILT Wageningen University and Research in the Netherlands. Figure 1 shows a schematic overview of the experimental design and a flow chart of the steps taken during the experiment.

#### Soil Column Setup

##### Soil Column Preparation

The experiment was conducted using a series of polyethylene (PE) hydraulic soil columns with a diameter of 12 cm and depth of 25.5 cm. The bottom of each column was covered with a thin nylon mesh with apertures (< 1 mm). Prior to the experiment, all soil types used (LSH, LSH, and Sand) were air dried at room temperature (for 7 days) and sieved using sieve #5 (apertures size was 4 mm) to keep the soil properties close to the initial soil.

Before filling the columns with soil, a 2-cm layer of fine gravel (2–4 mm) was added to the bottom (to work as a filter for soil particles from the column). Then, each column was filled up to 12 cm of soil representing three layers (each layer ± 4 cm). While adding the soil, the column wall was gently tapped to minimize air entrapment and to achieve homogenous soil packing. With the three lower soil layers in place, the columns were saturated with three pulses of RW. Finally (after 24 h), a top layer spiked with AgNPs was added as layer 1, ensuring that it had the same moisture content as the other soil layers in the soil column.

##### Rainwater Pulses

RW was applied to the top of the columns in pulses. Each pulse equaled 1 pore volume (PV) of the soil in the particular column. PV was determined from the total porosity of the soil samples which was considered to be equal to the volumetric soil water content at saturation. Therefore, to bring the soil in a column to the saturation level, a volume of water equal to the difference between dry moisture and the saturation level was necessary. The calculated porosities of the LSH and LSL soil columns were not significantly different. Thus, a fixed 42.2%, PV value equal to the average pore volume of these soils was used. To avoid problems related to inconsistent calculations of the AgNP concentration collected from different columns, this PV value was also applied to the Sand columns.

RW was applied using an inverted volumetric flask fixed above the column and connected to a sprinkler. The RW application rate was such that a constant amount of water was maintained on the top layer. Following addition of the AgNP-spiked soil to the columns, 1 RW pulse was applied per day during the experiment.

##### Bromide Tracer Breakthrough Check

To confirm that the column setup was functioning well, bromide (Br) was used as a soluble conservative tracer to test breakthrough volumes. Breakthrough data is an indicator of the hydrodynamic properties of the soil column.

**Fig. 2:**
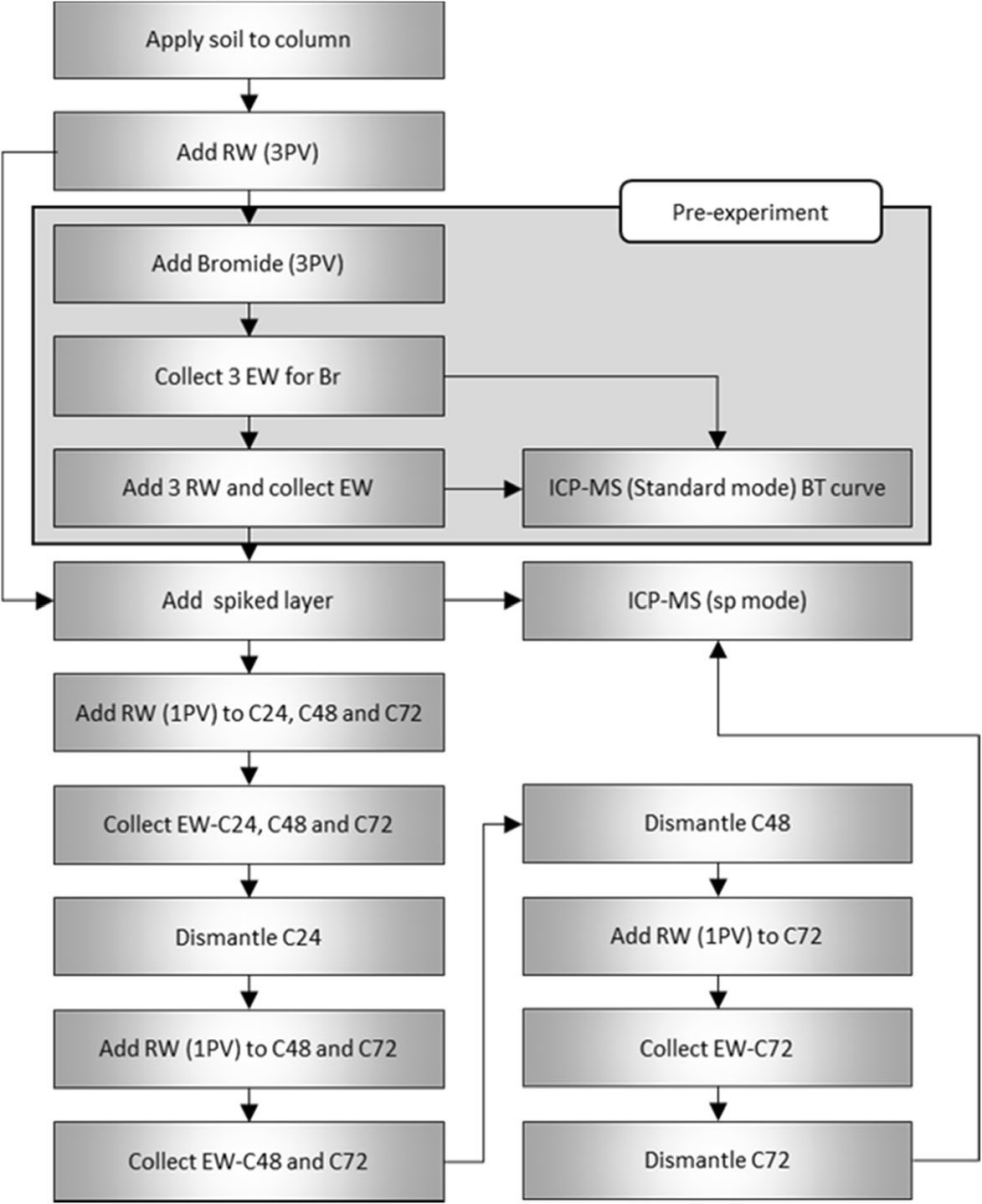
Schematic flow chart of the steps in the experiment. RW is rainwater; PV is pore volume; EW is effluent water; C is column series; the numbers 24, 48, and 72 depict the residence time in the column. The flow chart represents one replicate for one soil type

Three columns for each soil type were tested before the experiment. The columns were prepared in the same manner as described above with the exception that the top layer (layer 1) was not spiked with AgNPs. Each column was first saturated with 3 RW pulses. Then, three pulses containing 20 mg L^−1^ Br were applied followed by another three pulses of RW to remove all Br from the column. Bromide concentrations were detected using an inductively coupled plasma mass spectrometer (ICP-MS) in standard mode (Creed et al. 1996). A schematic flow chart depicting the steps during the experiment is shown in Fig. 2.

#### AgNP Application

The AgNP-spiked soil that was added to the columns (as layer 1) was prepared by diluting the AgNP stock solution to get a net concentration of 50 μg kg^−1^ with an average particle size equal to ± 60 nm. Milli-Q water (MQW) produced by an Ultrapure Water system from Millipore (Amsterdam, the Netherlands) was used for diluting AgNP spikes, collecting AgNP samples and preparing all other additional chemicals. The AgNP-spiked solutions (in volumes equal to 1 PV) were mixed with the dry-sieved soil (± 400 g) for soil layer 1 which was then saturated to match the saturation of the other soil layers in the column. As previously noted, the soil spiked with AgNPs was added as the top soil layer (layer 1) for each column. After the addition of this layer, the columns were left in the dark to equilibrate for 24 h.

#### Sample Collection and Characterization

##### Sample Collection

During the experiment, the collection of the effluent water (EW) containing leached AgNPs was started after the addition of each RW pulse to a column and continued for 24 h. For column series 1 (C24), only 1 volume of EW was collected. For column series 2 (C48), 2 EW volumes were collected and for column series 3 (C72), 3 were collected. EW samples were collected using dark-glass flasks and stored at room temperature.

Soil samples were collected from the dismantled columns. Column series 1 (C24) was dismantled after 1 RW pulse (24 h), column series 2 (C48) was dismantled after 2 RW pulses (48 h), and column series 3 (C72) was dismantled after applying 3 RW pulses (72 h).

Columns were carefully dismantled and sliced into the four layers 1 to 4, each 4 cm thick. Each individual soil slice was homogenized and 3 sub-samples < 10 g were collected for the determination of AgNPs. This resulted in 36 samples per soil type. For soil property determination, 3 samples per soil layer (weight of the sample < 50 g) were taken, totaling 12 samples per soil type.

Other samples such as RW, initial soil, and AgNP concentration control samples were measured simultaneously with the samples (soil and EW) containing AgNPs from the columns to get a clear image for AgNP concentration and particle size calculations. In total, more than 100 soil and liquid (EW or RW) samples were collected for each soil type for AgNP detection and soil characterization.

##### Soil Property Characterization

Soil characteristics were determined both before the experiments (Table 1) and after dismantling the columns. Soil texture for the three soil types was analyzed using a laser diffraction technique at the University of Leuven (Leuven, Belgium). Soil pH was measured using a portable probe (WTW pH 340) obtained from WTW company (Weilheim, Germany). CEC (Ca^+^2 and K^+^) were analyzed by NIRs (near infrared spectroscopy) based on 0.0166 M cobalthexamine trichloride (ISO 1994) in the Coimbra College of Agriculture (Coimbra, Portugal). IS was derived directly from the electrical conductivity (EC) using the formula of the linear approach method (aqion 2016). EC itself was measured using a portable probe WTW pH 340i (WTW, Weilheim, Germany). Organic matter (OM) content was measured according to the standard ASTM method (ASTM 2000).

##### AgNP Quantification

AgNP concentration and particle size characteristics in soil samples and EW were quantified. AgNPs in soil samples were first extracted using the aqueous extraction method as described by Mahdi et al. (2016). In EW, AgNPs were extracted by settling and dilution to remove the leached-out soil particles and OM.

After extraction, AgNP samples were measured for concentration and particle size using the single particle inductively coupled plasma mass spectrometry method (spICP-MS). The inductively coupled plasma mass spectrometer (ICP-MS) used to perform the spICP-MS was a Thermo Scientific Xseries-2 from (MA, USA). The instrument and technical settings for spICP-MS have been described previously by Peters et al. (2015). All measurements were performed at RIKILT Wageningen University & Research (Wageningen, the Netherlands).

#### AgNP Mass Balance Calculations

The mass balance for the AgNPs transported in the soil columns was calculated based on the initial amount applied to layer 1 of each column and the final AgNP content detected in the soil layers (1, 2, 3, and 4), and the effluent water after each rainwater application. The total mass balance was calculated for AgNP content after the third rain application for all soil layers. EW samples were expressed in micrograms and compared with initial AgNP contents applied.

## Results

### Soil Layer Characterization

After the experiment, each soil layer was used to assess the selected soil properties. The results shown in Table 2 indicate that the soil properties in all soil layers were more or less identical. So, no significant differences were found between the soil layers for pH, OM, CEC, and IS.

**Table 2:** Average soil characteristics in the soil column layers (*n* = 5) ± st. dev.

Soil	pH (KCl)	OM (wt%)	CEC (meq 100 g^−1^)		IS (mM)
			Ca^+2^	K^+^	
LSH-1	7.3 ± 0.3	3.2 ± 0.11	4.1 ± 0.14	0.128 ± 0.13	0.9 ± 0.06
LSH-2	7.4 ± 0.2	3.1 ± 0.10	3.4 ± 0.12	0.190 ± 0.09	1.2 ± 0.5
LSH-3	7.4 ± 0.1	3.2 ± 0.14	3.4 ± 0.02	0.185 ± 0.11	1.0 ± 0.14
LSH-4	7.3 ± 0.1	4.0 ± 0.05	3.3 ± 0.04	0.220 ± 0.11	1.3 ± 0.44
LSL-1	6.3 ± 0.1	1.3 ± 0.15	1.8 ± 0.09	0.054 ± 0.02	1.2 ± 0.10
LSL-2	6.4 ± 0.3	1.7 ± 0.17	1.5 ± 0.03	0.069 ± 0.07	1.1 ± 0.11
LSL-3	6.1 ± 0.1	1.7 ± 0.41	1.4 ± 0.01	0.071 ± 0.12	1.3 ± 0.08
LSL-4	6.2 ± 0.1	1.6 ± 0.36	1.4 ± 0.03	0.081 ± 0.18	1.4 ± 0.22
Sand-1	7.7 ± 0.3	0.6 ± 0.17	0.4 ± 0.11	0.014 ± 0.10	0.6 ± 0.06
Sand-2	7.2 ± 0.2	0.1 ± 0.15	0.6 ± 0.13	0.009 ± 0.14	0.5 ± 0.04
Sand-3	7.1 ± 0.1	0.2 ± 0.09	0.4 ± 0.05	0.008 ± 0.01	0.5 ± 0.05
Sand-4	7.5 ± 0.2	0.1 ± 0.15	0.3 ± 0.05	0.008 ± 0.03	0.4 ± 0.05

*LSH* loam soil with high organic matter, *LSL* loam soil with low organic matter, *Sand* sandy soil with no organic matter, *OM* soil organic matter, *CEC* cation exchange capacity, *IS* ionic strength, *numbers (1–4)* soil column layer (1 = top layer and 4 = bottom)

### Performance of Soil Columns

**Fig. 3:**
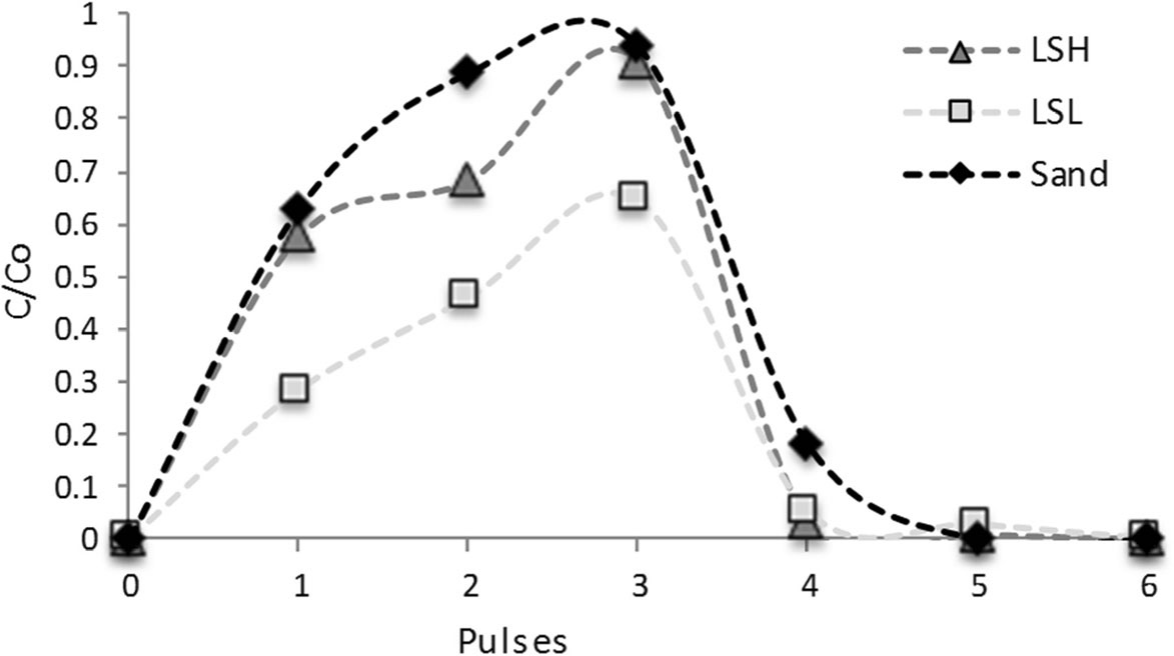
Breakthrough curves for bromide in the soil columns. LSH, column packed with loam soil with high organic matter; LSL, column packed with loam soil with low organic matter; Sand, column packed with sand. C/Co is ratio of the detected concentration of Br to the initially applied concentration. The first three pulses contained Br (20 mg L^−1^) and the others (4, 5, and 6) only rain water

The bromide tracer experiment revealed different breakthrough curves (see Fig. 3). Columns packed with sand showed the fastest and highest breakthrough and columns packed with LSL the slowest and lowest. Breakthrough curves of LSH showed an intermediate pattern.

### AgNP Transport in the Soil Columns

#### AgNP Concentration in the Soil Layers

**Fig. 4:**
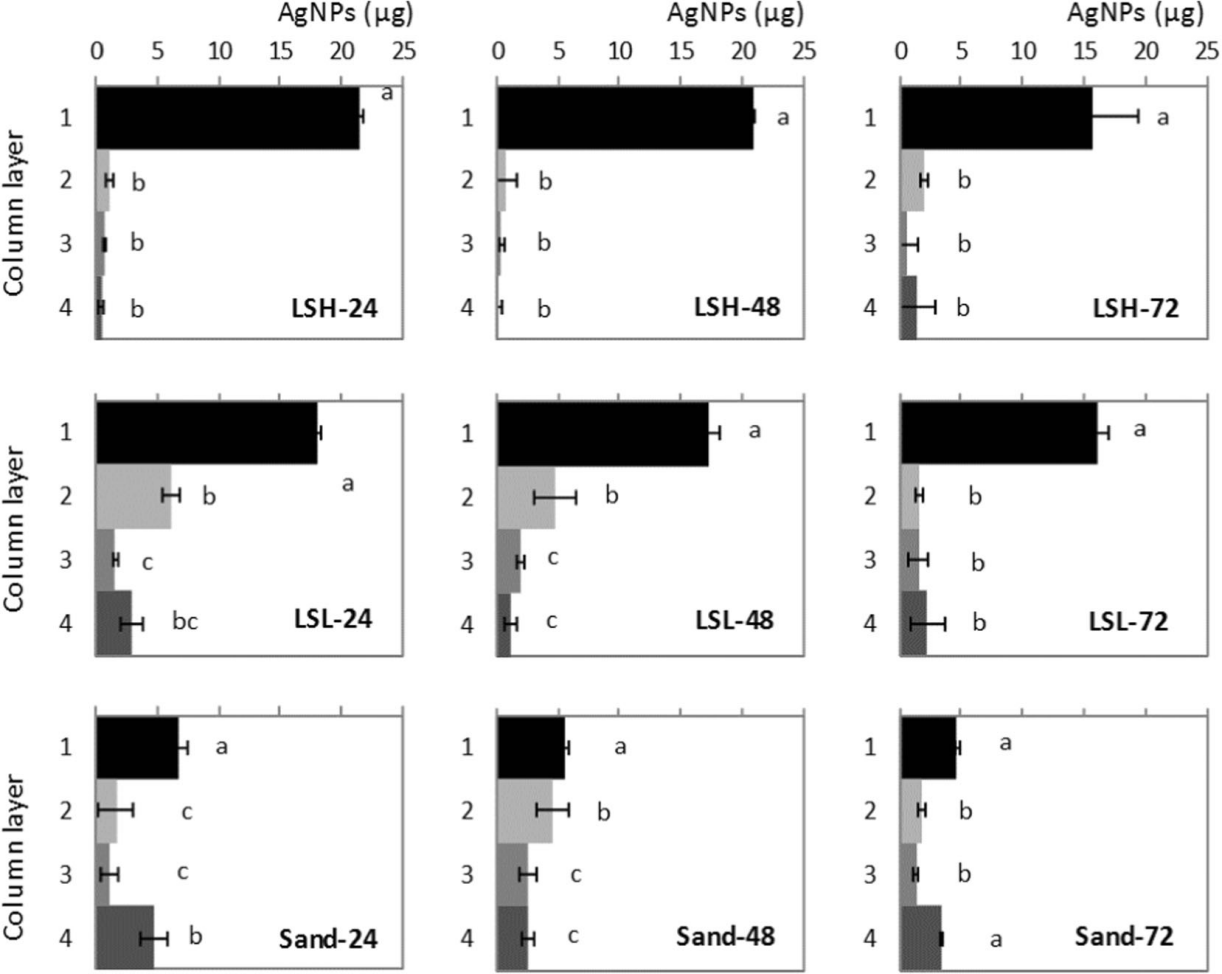
AgNP content in soil columns after the conclusion of the experiment. LSH, columns packed with loam soil with high organic matter; LSL, columns packed with loam soil with low organic matter; and Sand, columns packed with sandy soil; the numbers (24, 48, and 72) refer to the residence time of the AgNPs in the soil columns, and the time when they were dismantled to layers. Different lowercase letters indicate the significant differences (*p* < 0.05) in AgNP content between soil layers for the same soil column

The levels of AgNPs detected in the soil layers are shown in Fig. 4. In the columns packed with LSH, there was very limited AgNP transport after 24 h as less than 1 μg AgNPs was detected in each of the soil layers 2 and 3 and almost none in layer 4 while 20 μg of the initially applied AgNPs remained in layer 1. After 48 h, there was very little change in the transport of AgNPs compared with the 24 h results. However, after 72 h, AgNP content in layers 2, 3, and 4 increased; the detected amount in layer 2 increased to 2 μg, in layer 3 to 0.7 μg, and in layer 4 to more than 1.5 μg while AgNP concentration in layer 1 was reduced to 15 μg.

In the columns packed with LSL, the highest AgNP content was also found in layer 1. However, AgNPs detected in the soil layers 2, 3, and 4 were substantially higher than in the columns packed with LSH for all three sampling times (24, 48, and 72). The amount of AgNPs transported to the layers 2, 3, and 4 was the highest after 24 h and then decreased with time. Furthermore, AgNP content in layer 2 after 24 h was 6.2 μg, which was higher than the AgNP content in layers 3 and 4. However, after 72 h, AgNP content in layer 2 decreased to just 1.6 μg, which was lower than the AgNPs detected in layer 4.

In columns packed with sand, the AgNP transport pattern was different than that found in the LSH and LSL columns. In the Sand columns, layer 1 retained fewer AgNPs indicating that more AgNPs were transported to the deeper layers. After 24 h, AgNP content in layer 1 was 6.8 μg and in layer 4, it was 4.8 μg while in layers 2 and 3, the content was 1.6 μg and 1.1 μg, respectively. After 48 h, AgNP content in layers 2 and 3 increased to 4.5 μg in layer 2 and 2.6 μg in layer 3 while it dropped to 5.5 in layer 1 and 2.5 in layer 4. After 72 h, AgNP content in all layers decreased except in layer 4: in layer 1, it was < 5 μg; in layers 2 and 3, it was < 2 μg; while in layer 4, it was 3.4 μg. Furthermore, in several soil columns, we noticed a significant difference between AgNP content in layer 4 and layers 2 and/or 3. This is due to AgNP filtration problems from soil to gravel layers which was also the case in real scenarios (Kumahor et al. 2016).

#### AgNP Content in the Effluents

**Fig. 5:**
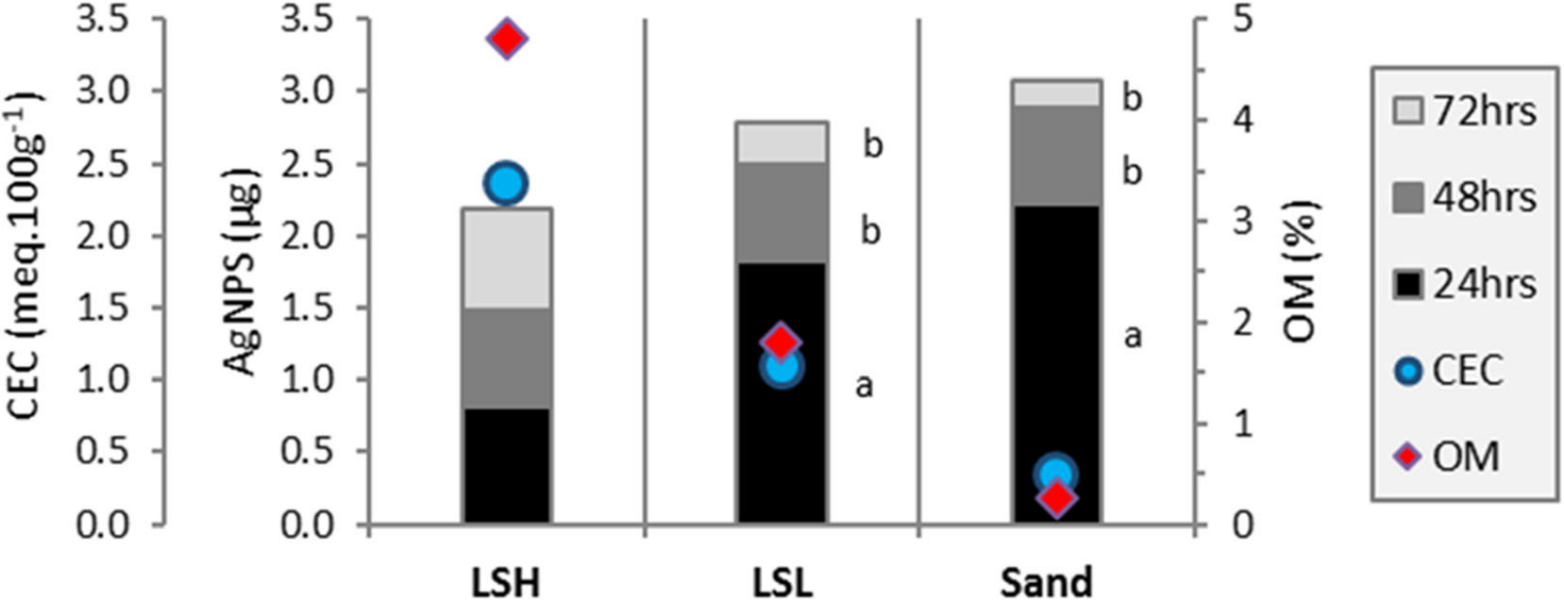
AgNP content detected in effluent water. LSH, columns packed with loam soil with high organic matter; LSL, columns packed with loam soil with low organic matter; Sand, columns packed with sandy soil; OM, organic matter (represented by the secondary *Y*-axis); and CEC, cation exchange capacity calculated for Ca^+2^ and K^+^. Different lowercase letters indicate the significant differences (*p* < 0.05) in AgNP content in the three collection times of EW

The results for the levels of AgNPs detected in effluent water (EW) are shown in Fig. 5. In the EW from columns packed with LSH, less than 1 μg of AgNPs was detected for each of the three collection times (24, 48, and 72 h). The total AgNP content in EW from these columns (2.2 μg) was the lowest of the totals from all three soil types.

For columns packed with LSL, AgNP content detected in the EW collected was highest after 24 h (± 1.8 μg) and then decreased significantly in the EW collected at 48 and 72 h, with only ± 0.3 μg detected at 72 h.

Similar to the results for the EW from the LSL columns, the AgNP content in EW from the Sand columns was highest after the 24-h collection and then decreased in the EW collected after 48 and 72 h. However, the amount detected after 24 h was greater than that detected in the LSL EW, making the total AgNP content collected from the sand EW the highest among all soil types.

Overall, the AgNP content in the EW increased from fine textured to coarse textured soil. As expected, there was an inverse relationship between OM and the AgNPs detected in the EW. A similar relationship existed for CEC.

#### Particle Size Tracking in the Columns and Effluent

**Fig. 6:**
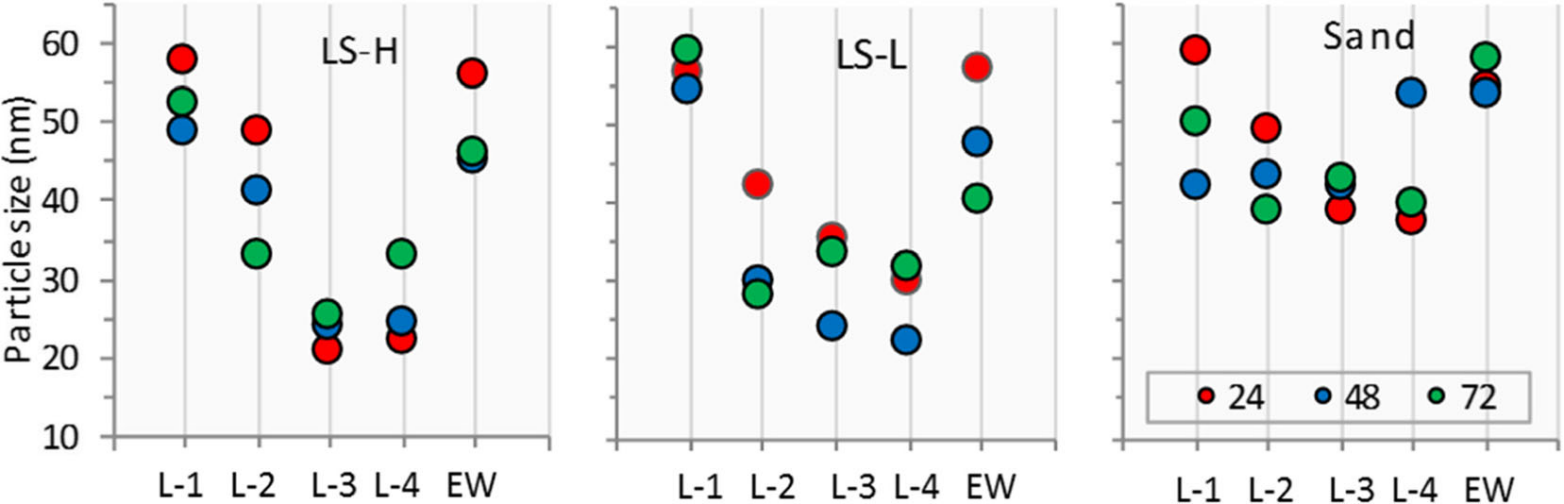
AgNP particle size in the columns. LSH, columns packed with loam soil with high organic matter; LSL, columns packed with loam soil with low organic matter column; sand, columns packed with sandy soil. (L-1 to L-4), soil layers; EW, column effluent; the numbers (24, 48, and 72) refer to the time of soil column dismantling and the time to collect the effluent

The particle size of the AgNPs decreased continuously in the soil column layers as particles moved from the top to the bottom layers as shown in Fig. 6. This was true for all soil types, although the amount of change differed between soils. In the LSH and LSL columns, AgNP particle size in layers 3 and 4 was reduced by 60–70% after 72 h of AgNP residence time in the columns. In Sand columns, the particle size reduction was 40–50% after 72 h.

In the EW, AgNP particle sizes are generally larger than the ones found in soil layers 2 to 4, indicating the existence of flood paths facilitating rapid transport of particles from layer 1 to the effluent water. In the Sand columns, particle size decreased the least.

### Total AgNPs Transported Through the Soil Columns

**Fig. 7:**
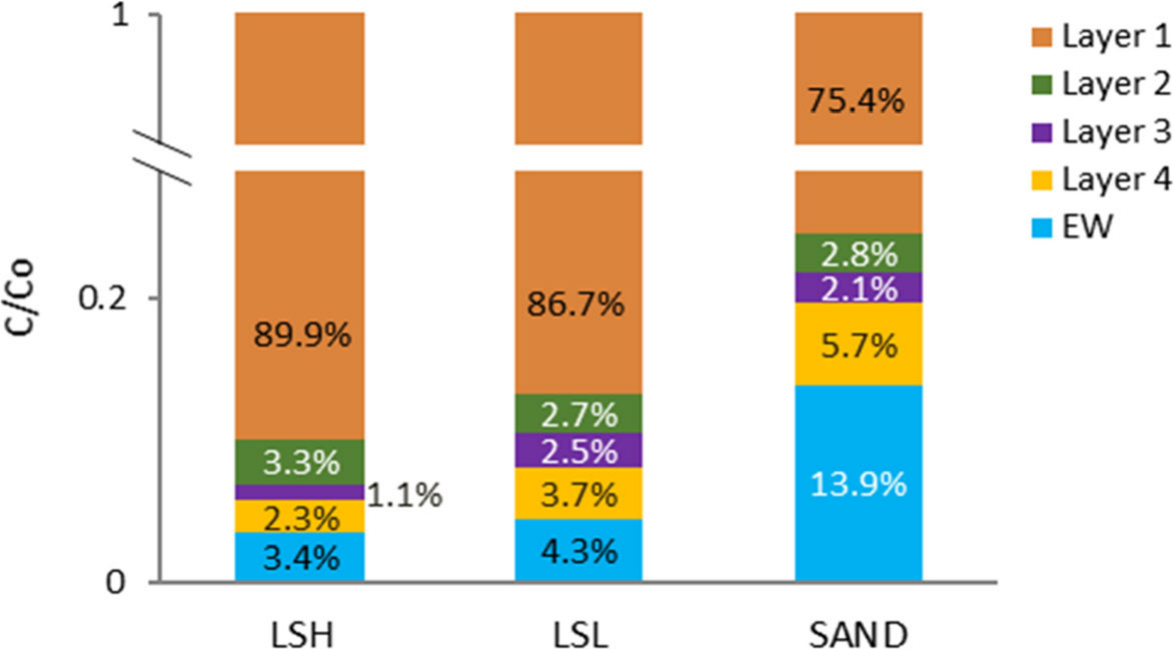
Total transported amount of AgNPs in the soil columns’ layers and in the effluents. C/Co is the ratio of the initial applied AgNP concentration to the detected concentration; LSH, columns contain loam soil with high organic matter; LSL, columns contain loam soil with low organic matter column; Sand, columns contain sandy soil. EW, the column effluents accumulated for the three times of collection

As shown in Fig. 7, the total amounts of AgNPs transported from layer 1 to layers 2–4 and EW were 10.1%, 13.3% and 24.6% of the initial applied AgNPs in the LSH, LSL, and Sand columns respectively.

## Discussion

### AgNP Distribution in the Soil Layers

AgNP transport patterns differ per soil and per layer as a result of the differences in OM, CEC, and soil texture.

In the columns packed with LSH, slow AgNP content transport was noticed during the first two collections (24 and 48 h). However, after 72 h, the amount of AgNPs detected had increased in all layers, especially in layers 2 and 4. This slow transport was attributed to the curtailment of AgNP transport due to the fine texture of LSH (compared to LSL and Sand) and to the related high organic matter and CEC content. Fine textured soil with high values of OM and CEC have the potential to increase AgNP adsorption on soil particles (Sagee et al. 2012; Coutris et al. 2012; Liang et al. 2013a).

In the columns packed with LSL, faster AgNP transport (than in LSH columns) was noticed after applying the first RW pulse (samples collected after 24 h), causing relatively high concentrations in deeper soil layers and effluent water. This was due to the fact that LSL is less finely textured than LSH, which reduced the adsorption potential of AgNPs on soil particles, thus increasing AgNP mobility (Shoults-Wilson et al. 2011b). The lower OM and CEC in LSL increased AgNP mobility as OM can inhibit AgNP mobility by coating the Ag particles and the lower CEC content can reduce cation bridging (Liang et al. 2013a). After 48 and 72 h, only small differences in the AgNP content of layers 2 and 3 were noticed and the layers had almost the same AgNP content after 72 h.

In the columns packed with sand, the AgNP transport pattern was fast and unstable due to the coarse texture of the sand. Our findings confirm what has been reported by others (Sagee et al. 2012; Liang et al. 2013b). In addition, there was no diminished AgNP transport due to low OM, CEC, or IS content.

Therefore, AgNP retention and/or mobility mechanisms inside the soil profile can be affected by the physical properties of soil such as particle size distribution and soil chemistry, like IS, OM, CEC, and pH. When soil particle sizes decrease, their surface area to volumes will increase which increase the attachment of AgNPs on these soil particles. Increasing attachment to soil particles means that AgNPs would experience restricted mobility and increasing effects from OM, CEC, IS, and pH. The existence of high OM would restrict the mobility of AgNPs due to increasing of AgNP attachments on OM. High CEC, as confirmed in this study, was found to have a stronger effect when AgNP particle size decreased. Regarding pH, we maintained a neutral pH in all soils used so that the mean pH would not play a role in the understanding of retention and mobility mechanisms of AgNPs inside soil (Yin et al. 2015a). IS values, on the other hand, were the lowest in sand-packed columns. This enhanced AgNP mobility and transport due to the compression of the electric double layer (Liang et al. 2013a).

### AgNP Concentration in the Effluent Water

As with the distribution of AgNPs in the soil layers, AgNP content in EW was influenced by soil texture, OM, and CEC. As a result, coarsely textured media with a low OM content allowed the most rapid and distinct transport of AgNPs towards the bottom of the columns and resulted in high concentrations in effluent water.

### Particle Size Distribution

AgNP particle sizes in the soil layers and in the EW were also monitored during the transport process. The particle size of AgNPs has been linked to the potential toxicity impact of AgNPs in many studies, with smaller particles expected to have a higher toxicity (Pal et al. 2007; Kim et al. 2012; Silva et al. 2014). In general, our results showed that AgNP particles became smaller as they moved through the soil over time and their new size depended on the soil type and soil depth. Figure 6 shows some differences in the AgNP sizes inside each individual column for layers 2, 3, and 4 but they were not significant. However, particle size differences for AgNPs retained in these layers and the AgNPs in layer 1 and in EW were significant.

In the columns packed with LSH and LSL soils, AgNP particle sizes decreased with time and depth due to chemical interactions with the soil particles. In the columns packed with sand, the AgNP particle sizes were more stable than in the LSH and LSL columns. While most of the particle size decreases occurred in the lower layers, some also occurred in layer 1. The AgNP concentration in layer 1 was far higher than in the other layers so the dissolution of AgNPs was less.

Yin et al. (2015a) reported that AgNP particle size decrease was related to the AgNP properties (initial concentration, coating, particle size, etc.) and to the receiving soil medium (pH, IS, CEC, OM, soil type, and soil particle distribution). In the current study, only one type and one particle size of AgNPs was used so differences in AgNP particle sizes in this study are expected to have been the effect of soil characteristics. In general, AgNP particle size decrease occurs directly after AgNPs is mixed with water due to the formation of Ag_2_O which enhances the Ag^+^ release (Sotiriou et al. 2012). Our results showed that Ag particle size in the soil layers was subjected to more dissolving than in the EW. The reason for this is that AgNP mobility in the columns packed with LSH and LSL soils was restricted due to the presence of OM and larger amounts of the fine-textured particles (clay and silt) compared to sand. This caused the AgNPs to be trapped in the pore voids of soil. This trapping also led to increased interaction between AgNPs and soil particle reactive compounds like OM and CEC.

In EW, AgNPs were transported “mostly” directly from layer 1 (where the AgNPs were applied) to the outlet which means that CEC and OM had less of an effect on particle size than what occurred in the soil layers. This was verified by following the time scans of Ag^+^ signals in ICP-MS. In the time scan for layers 2, 3, and 4 of all columns, where the AgNP particle size decrease was higher than in layer 1, the higher signal of Ag^+^ meant that there was a higher rate of dissolution. However, in layer 1 and the EW, the AgNP size decreased less and the Ag^+^ signal was lower. This verifies that AgNPs in EW were mostly leached directly from layer 1.

## Conclusion and Recommendations

- AgNPs have been confirmed to be transported with water in soil columns which can lead to retention in deeper soil layers and leaching via effluent water despite the low (initial) concentrations applied. This may cause pollution.
- The detection of AgNPs in soil layers and in the effluents confirms the potential risk of contamination resulting from AgNP application to soil.
- AgNP retention in soil was affected by soil OM and AgNP particle size was affected by the CEC of soil.
- The spICP-MS method allowed for the detection of AgNP particle size in soil layers and in the effluents, which revealed that AgNPs in the effluent were more stable in relation to size than in the soil layers which may impact water bodies.
- Apart from the fact that AgNPs can be transported through soils, they may also be redistributed by runoff and erosion processes in undulated areas due to the strong adsorption of AgNPs to soil particles.
- Further research on the transport of AgNPs in soil is needed focusing on lower concentrations of applied AgNPs, other AgNP particle sizes and other types of soil to assess the differences in transport dynamics and potential toxicity of the transported AgNPs on different soil species and plants.
